# Healthy Parent Carers: feasibility randomised controlled trial of a peer-led group-based health promotion intervention for parent carers of disabled children

**DOI:** 10.1186/s40814-021-00881-5

**Published:** 2021-07-23

**Authors:** Gretchen Bjornstad, Beth Cuffe-Fuller, Obioha C. Ukoumunne, Mary Fredlund, Annabel McDonald, Kath Wilkinson, Jenny Lloyd, Annie Hawton, Vashti Berry, Mark Tarrant, Aleksandra Borek, Katharine Fitzpatrick, Annette Gillett, Shelley Rhodes, Stuart Logan, Christopher Morris

**Affiliations:** 1 Peninsula Childhood Disability Research Unit (PenCRU), University of Exeter Medical School, University of Exeter, St. Luke’s Campus, Heavitree Road, Exeter, EX1 2LU UK; 2 NIHR Applied Research Collaboration South West Peninsula (PenARC), University of Exeter Medical School, University of Exeter, St. Luke’s Campus, Heavitree Road, Exeter, EX1 2LU UK; 3 Relational Health Group, Institute of Health Research, University of Exeter Medical School, University of Exeter, Exeter, EX1 2LU UK; 4 Health Economics Group and NIHR Applied Research Collaboration (PenARC) South West Peninsula, University of Exeter Medical School, University of Exeter, Exeter, EX1 2LU UK; 5 Nuffield Department of Primary Care Health Sciences, Medical Sciences Division, University of Oxford, Radcliffe Observatory Quarter, Woodstock Road, Oxford, OX2 6GG UK; 6 Exeter Clinical Trials Unit, University of Exeter Medical School, University of Exeter, St. Luke’s Campus, Heavitree Road, Exeter, EX1 2LU UK

## Abstract

**Background:**

Parent carers of children with special educational needs or disability are at higher risk of poor mental and physical health. The need for a tailored, peer-led group programme was raised by parent carers, who co-developed the Healthy Parent Carers programme with researchers. This study aimed to test the feasibility of programme delivery in community settings, and the feasibility and acceptability of a randomised controlled trial design.

**Methods:**

Participants were individually randomised with concealed allocation to a structured group programme and access to online resources (intervention), or access to the online resources only (control). Measures of wellbeing and secondary and economic outcomes were collected before randomisation, immediately post-intervention, and 6 months post-intervention. Descriptive statistics on recruitment and attrition, demographics, attendance, and fidelity of intervention delivery were analysed with feedback on the acceptability of the trial design.

**Results:**

One hundred and ninety-three parent carers expressed an interest in taking part. Ninety-two participants recruited from across six sites were randomised (47 intervention, 45 control). Lead and assistant facilitators were trained and delivered the group sessions. Sixteen (34%) participants in the intervention arm did not attend any sessions, and attendance varied across sites and sessions. One participant withdrew post-randomisation, and 83 (90%) participants completed outcome measures at the six-month follow-up.

**Conclusions:**

The study demonstrated that it was feasible to deliver the programme in community settings. The number of parent carers who expressed interest signifies the need for such a programme and the feasibility of recruiting to a definitive trial. Loss to follow-up was low. Further research is needed to explore ways to reduce barriers to participation in person and assess the feasibility and acceptability of programme content and delivery for more ethnically diverse groups, and potentially using interpreters. Given the Covid-19 pandemic and delivery format feedback, there is also a need to investigate remote or blended delivery strategies. Although the results indicate that a definitive trial is feasible, programme impact would be strengthened through exploration of these uncertainties.

**Trial registration:**

ISRCTN, ISRCTN15144652, registered on 25 October 2018, ClinicalTrials.gov, NCT03705221, registered on 15 October 2018.

**Supplementary Information:**

The online version contains supplementary material available at 10.1186/s40814-021-00881-5.

## Key messages regarding feasibility

- What uncertainties existed regarding the feasibility?
  - ◦ The Healthy Parent Carers programme was developed using intervention mapping and was initially tested with one group of seven parent carers. Following this, the intervention content and delivery methods were refined based on feedback from programme facilitators and participants.
  - ◦ Further testing was needed to investigate whether it can be feasibly delivered in community settings by trained facilitators who had not been involved in the development of the programme.
  - ◦ Testing the design for a randomised controlled trial to determine whether a definitive trial is likely to be acceptable to participants and feasible to evaluate the effectiveness and cost-effectiveness of the programme.
- What are the key feasibility findings?
  - ◦ Six venues in a variety of community settings were established for group delivery by trained facilitators.
  - ◦ A sufficient number of participants were recruited in each study site and randomised to groups in the intervention arm (total N=92).
  - ◦ According to facilitators’ self-reports, 90% of programme activities were delivered across all groups.
  - ◦ Participant attendance was variable, with two groups having consistently low numbers attending, whereas others had higher levels of attendance.
  - ◦ Outcome data were collected from 91% of participants at post-intervention follow-up and 90% of participants at 6-month follow-up.
  - ◦ Most participants found the trial design to be acceptable, although some would have preferred to choose the mode of programme delivery.
  - ◦ The outcome measures were largely acceptable to participants, although some technical problems with the electronic patient-reported outcomes system were reported.
  - ◦ The cost-effectiveness framework was found to be feasible for implementation in a trial.
- What are the implications of the feasibility findings for the design of the main study?
  - ◦ There was considerable interest in the study from parent carers, indicating that the recruitment strategy could be effective for recruiting sufficient numbers to trial sites.
  - ◦ Social media and events were found to be the most helpful elements of the recruitment strategy.
  - ◦ The trial design was generally acceptable to parent carers. Clearer information about allocation in adverts may help to manage expectations. The electronic patient-reported outcomes system should be revisited and re-tested by users before implementation in a definitive trial.
  - ◦ Retention and participant response to follow-up was successful utilising a combination of automated email reminders, phone calls, text messages and vouchers for acknowledgements. This system should be replicated in a definitive trial to ensure similar follow-up rates.

## Background

As of January 2019, there were an estimated 1,320,000 school-aged children in England with special educational needs, which is 14.9% of all pupils [1]. Around 1.1 million children (8%) in the UK have a disability, according to the Family Resources Survey 2018/2019 [2]. Parent carers of children with special educational needs or disability (SEND) provide care throughout their childhood, through their transition to adult services, and sometimes beyond. Over the past several years, many international studies have consistently shown that this long-term caring role puts parent carers at greater risk of mental health problems, particularly stress and depression, and physical health problems, compared with other parents [3–17]. These problems may worsen over time and affect parent carers’ ability to care for their children [18]. However, parent carers’ experiences vary and are not necessarily related to the complexity of their child’s disability [5, 6], with some reporting positive impacts [9, 19].

The need for an intervention specifically designed to address this risk was raised by parent carer members of the Peninsula Childhood Disability Research Unit (PenCRU) Family Faculty public involvement group. These parent carers worked closely with researchers over several years to co-create the Healthy Parent Carers (HPC) as a peer-led, group-based programme. The programme was developed iteratively and systematically using the Intervention Mapping approach [20]. A proof-of-principle study with one group of seven parent carers indicated that the intervention was feasible to deliver in a university setting by facilitators involved in its development [21]. It was also found to be acceptable to and valued by parent carers. The facilitator’s intervention delivery manual and programme content were revised following feedback.

Before progressing to a definitive randomised controlled trial, it was essential to assess the feasibility of delivery of the programme by newly trained facilitators outside of the intervention development team in a range of community settings in several groups at one time and to assess the feasibility and acceptability of trial processes [22].

## Methods

### Aims

This trial aimed to evaluate whether the programme can be delivered in the community by newly trained facilitators and whether a randomised controlled trial design with cost-effectiveness analysis is feasible and acceptable to participants.

This paper focuses on the assessment of the following objectives:

- Feasibility of establishing venues and recruiting and training facilitators to deliver the intervention
- Feasibility of recruiting participants in different sites
- Acceptability of trial processes to parent carers
- Fidelity of intervention delivery in terms of format, content, and quality
- Attendance in the group programme
- Loss to follow-up and estimates of standard deviations for the outcomes to help inform the sample size calculation for the definitive trial
- Feasibility of the proposed cost-effectiveness framework for a future definitive trial

The acceptability of the intervention and training for facilitators was also assessed, but results are reported separately in order to present adequate details (Lloyd J, Bjornstad G, Borek A, Cuffe-Fuller B, Fredlund M, McDonald A, Tarrant M, Berry V, Wilkinson K, Mitchell S, et al: The Healthy Parent Carers programme: mixed-methods process evaluation and refinement of a health promotion intervention, Under review).

Criteria for progression to a definitive trial were set a priori and were as follows:

a) Recruit a minimum of 48 participants, which was the minimum number to enable all six sites to be randomised and the intervention to be tested.

b) Six intervention groups delivered in the intervention arm, assessed by establishing 6 venues, and identifying and training facilitators, and groups completing the programme curriculum.

c) At least 80% of participants completing measures at 6-month follow-up or a clear plan to achieve this in the trial.

### Study design

**Fig. 1:**
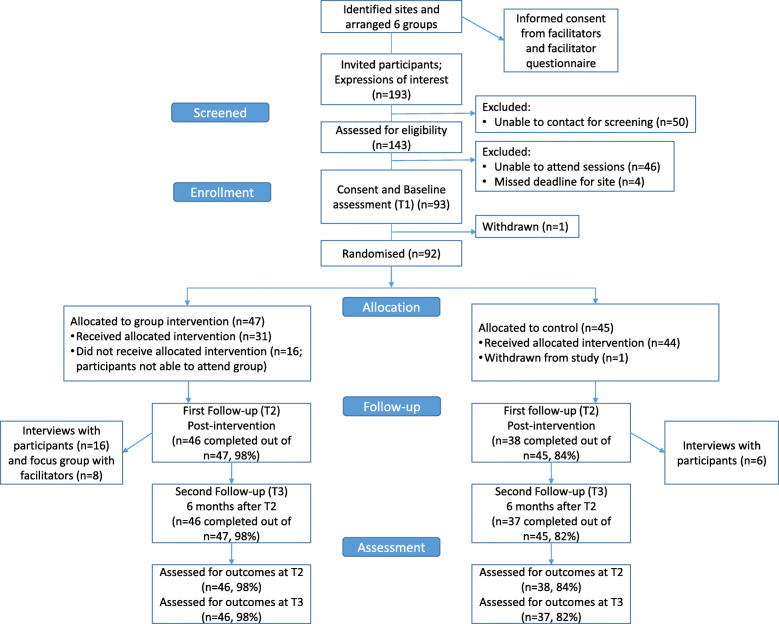
CONSORT flow diagram

A feasibility study using a parallel group randomised controlled trial design and within-trial cost-effectiveness analysis was carried out in six sites in the South West of England. Participants were randomly allocated to receive the group-based HPC programme and access to online programme resources (intervention arm) or to receive access to the online resources only (control arm). Participant feedback forms, participant interviews, facilitator questionnaires and feedback forms, facilitator checklists, and a facilitator focus group provided data for the process evaluation. Outcome data collection took place at three time points in both trial arms: at baseline (prior to randomisation), immediately post-intervention, and 6 months later. The trial design and the flow of participants through the trial are illustrated in the CONSORT Extension to Pilot and Feasibility Trials flow diagram (Fig. 1). The CONSORT reporting checklist is provided as an additional file (see Additional file 1). The methods were provided in detail in the protocol [23].

### Public involvement

This project has involved parent carers and stakeholders from inception to ensure that (a) the research is conducted in an acceptable manner, (b) the research outputs are relevant and useful to parents of children with special educational needs and disabilities, and (c) our dissemination materials and methods are appropriate and accessible.

Over 40 parent carers from the PenCRU Family Faculty public involvement group have been involved in all stages of developing the HPC programme and designing and conducting the feasibility trial. During this trial, parent carers met with the research team for a half-day meeting at least once per school term. They were also involved in additional ways, such as volunteering to review and provide feedback on the online programme resources, the recruitment advertisements and participant information sheets, and the outcome measures used in this study. They continued to meet to interpret the results, disseminate findings, and plan subsequent stages of the research. Two parent carers were co-investigators of this trial, delivered the training, and are co-authors of this paper (MF and AM).

Our Stakeholder Advisory Group (SAG) included 20 representatives from the Local Authority, Public Health, four parent carer forums in south west England, relevant charities, and special schools. This group met four times over the course of the study to provide input on the design of the study, the establishment of programme delivery sites, and facilitator and participant recruitment.

### Establishing sites

Six venues were established in the south west of England. These varied in nature to assess the suitability of programme delivery in a variety of settings. They comprised two special schools, one children’s hospice, one community adult learning centre, one parent carer forum office, and one community hotel that regularly provides a venue for local parent carer groups and other community events. All venues met requirements for delivery of the group sessions in terms of accessibility (both within the building and in terms of parking and/or public transport), room size, availability of Wi-Fi and a projector and screen, toilets, facilities to make tea and coffee, and cost. Days, times, durations, and frequencies of sessions were agreed with each venue. The six venues also varied in terms of location (north or south Devon, Cornwall, and Somerset) and geography (cities or towns).

### Recruitment and training of facilitators

Lead Facilitators were recruited by referral via the Council for Disabled Children (CDC). The CDC runs a training programme for parent carers called the ‘Expert Parent Programme’, which is delivered by parent carers. Three of these parent carers were selected as being among the most experienced at delivering group interventions to parent carers and being available to deliver groups in the south west of England.

Assistant Facilitators were recruited through adverts shared by members of the SAG to contacts in the six delivery areas in July–September 2018. Adverts included information about the role and a person specification which included criteria such as being a parent carer, having current knowledge and understanding of how being a parent carer can impact on health and wellbeing, the ability to work with parents in a sensitive and empathic way, upholding confidentiality at all times, being non-judgemental, and being available to attend scheduled train-the-facilitator sessions and weekly group sessions. They were not expected to have experience delivering interventions. Applicants were interviewed by a researcher by telephone and selection decisions were made by the research team based on applications and interview notes. The aim was for the Assistant Facilitators to be local to the group site; however, this was not always possible with distances from the facilitator/s’ home to the venue ranging from 12 to 40 miles.

All Lead and Assistant Facilitators provided written informed consent before beginning training. Facilitator training was led by two parent carers who co-developed the programme and facilitated the group in the previous study (MF and AM), supported by researchers [21]. The training was structured and documented in a training manual. Three Lead Facilitators received the first 2 days of training. After this initial training, it became clear that one of these facilitators would be unable to deliver group sessions due to travel logistics. Two Lead Facilitators attended the subsequent 2 days of training along with six Assistant Facilitators and three reserve Assistant Facilitators. A further day of training for all facilitators was provided as a refresher after the first two groups completed all sessions and before the next four groups started sessions.

### Participant recruitment

Recruitment took place in two sites in one school term (October–December 2018) and in four further sites in the subsequent two terms (January–May 2019). Press releases and local television and radio interviews/online features in newspapers were used to announce the study. Members of the SAG shared the study advert with their contacts and networks. The advert and information about the study was also shared on our social media sites, with special schools and Special Educational Needs Coordinators (SENCOs) in mainstream schools, Information and Advice Services, advisory teaching services, disabled children’s services, and other contacts such as parent carer groups in the south west of England. Members of the research team also attended events hosted by parent carer forums and groups, where they shared information about the study, with opportunities for parents to discuss the study with staff individually if they wished.

Screening was conducted by telephone or in person at recruitment events by research staff. Inclusion criteria were (1) primary carers of children with additional needs or disability (participants who self-identify as primary carers were eligible; the child could be up to 25 years old consistent with the current Department of Health and Department of Education Special Educational Needs and Disability (SEND) legislation in England and The Children’s Act; no named diagnosis was necessary, and we did not limit to specific conditions), (2) willing and able to attend the programme group meeting session(s) on arranged dates/times, and (3) able to access online information. Only one parent per household were able to take part as individuals were randomised. Written informed consent was provided in person in individual meetings with research staff.

We aimed to recruit 96 participants (16 per site on average). This is large enough to estimate the standard deviation for continuous outcomes in each arm within 29% of its true value based on the upper bound of the 95% confidence interval, if 80% of the sample provide data at follow-up. A minimum of eight participants per site needed to be recruited to ensure that at least four participants would be allocated to each of the six HPC programme delivery groups within the intervention arm.

### Participant allocation

A computer-generated randomisation sequence was used to allocate participants on a 1:1 ratio to intervention and control conditions. A block randomisation scheme was implemented to ensure equal numbers of participants were allocated to each trial arm, stratified by group delivery site. The allocation sequence was concealed from researchers using a randomisation service set up and maintained by the Exeter Clinical Trials Unit. Blinding was not used in this trial.

Participants allocated to the intervention arm were sent details of the group sessions and contacted by phone by the Lead Facilitator of their group. Participants in both the intervention and control arms received a link to the online programme resources and instructions on the webpage.

### Intervention

The intervention arm involved the peer-led, HPC programme delivered in groups, as well as access to online resources. Full details of the intervention, including its development, logic model, and content (e.g. activities, behaviour change techniques), are available [21]. The programme aims to foster a sense of shared social identity as members of the programme group, enable social support from fellow group members, and promote parent carers’ confidence, motivation, and empowerment to take steps to improve their health and wellbeing (i.e. engage in health-promoting activities). These components may cultivate the conditions for change necessary for individuals to feel able to make their own plan to prioritise healthy behaviours in ways that are feasible and important to them [24].

The programme content is based around a set of universal and evidence-based actions (called CLANGERS) associated with health and wellbeing. CLANGERS stands for Connect, Learn, be Active, Notice, Give, Eat well, Relax, and Sleep [25]. The ‘CLANG’ component comprises the ‘Five Ways to Wellbeing’ based on the evidence from the Foresight project on Mental Capital and Wellbeing [26]. Each of these behaviours is potentially more difficult for parent carers.

The programme consists of 12 modules. The modules can be delivered in groups over 6 weekly sessions (comprising two modules per session lasting four hours) or 12 weekly sessions (one module per session lasting 2 h). In this study, the 6-session model was delivered in 5 sites in the daytime, and the 12-session model was delivered in one site in the evenings.

The online resources are organised into the same 12 modules, each with a text document to read (approximately 3 pages) and one or more video or audio files with content illustrating the module topic and providing information. Participants in the intervention arm received a link and password to the site for their group, and the materials were added on a weekly basis in line with their group’s progress through the modules.

Each HPC group was delivered by one Lead Facilitator and one Assistant Facilitator. One Lead Facilitator delivered two groups and the other delivered four groups. Each group had a different Assistant Facilitator.

### Comparison

Participants in the comparison arm were given access to the online resources for the programme only. These were provided on a password-protected website with the resources for all modules available at once, to progress at their own pace.

### Outcome measures

All participants were asked to complete measures at three time points in both trial arms: at baseline (prior to randomisation), immediately post-intervention for their group (dates ranged from April–July 2019, and 6 months later (September 2019–January 2020). Measures were completed using an online platform maintained by the Exeter Clinical Trials Unit. Wellbeing was measured using the Warwick-Edinburgh Mental Wellbeing Scale (WEMWBS) [27]. Mental health was assessed using the Patient Health Questionnaire-9 (PHQ-9) [28–30]. If a participant scored higher than 0 on question 9 on the PHQ-9 (‘Thoughts that you would be better off dead or of hurting yourself in some way’), this was recorded, and a member of the research team followed the study safeguarding protocol.

The Health Promoting Activities Scale (HPAS) was used to measure the frequency of participation in activities to promote health [31, 32]. The Patient Activation Measure (PAM) was used to measure participants’ skills, knowledge, and confidence in managing their own health [33, 34]. Protective factors such as resilience, social connections, and practical support were measured using the Parents’ Assessment of Protective Factors (PAPF) [35].

The EuroQol 5 Dimensions (EQ-5D-5L) was used to measure health-related quality of life [36]. The ICEpop CAPability measure for Adults (ICECAP-A) was used to measure the following aspects of wellbeing: attachment, stability, achievement, enjoyment, and autonomy [37–39]. Participants’ use of health care, social care, and wider societal resources was measured using a study-specific resource use questionnaire. Full details of the measures used are in the study protocol [23].

Participants received a £25 shopping voucher for completing measures at each time point. Participants were contacted to complete post-intervention and follow-up measures by automated email reminders, with phone calls and text messages from researchers if required.

### Cost-effectiveness framework

As part of the feasibility study, a framework was developed and tested for assessing the cost-effectiveness of the intervention in a future randomised trial. This included:

a) Establishing methods for estimating intervention resource use and costs (e.g. training of facilitators, facilitators’ time, venue hire), in collaboration with the programme facilitators and site representatives;

b) Developing a health, social and wider care service resource use questionnaire in collaboration with parent carers, drawing on measures in the Database of Instruments for Resource Use Management (DIRUM) repository [40];

c) Assessing the feasibility of the EQ-5D-5L [41] and the ICECAP-A [42] for use with parent carers, for use in estimating quality-adjusted life-years (QALYs) and wellbeing-adjusted life-years (WALYs).

### Process evaluation data

A process evaluation ran alongside this trial to understand delivery issues and experiences of delivering or participating in the programme. Qualitative and quantitative data were collected to address the process evaluation questions addressed in this paper as described below. Further process evaluation questions, methods, and results are reported separately (Lloyd J, Bjornstad G, Borek A, Cuffe-Fuller B, Fredlund M, McDonald A, Tarrant M, Berry V, Wilkinson K, Mitchell S, et al: The Healthy Parent Carers programme: mixed-methods process evaluation and refinement of a health promotion intervention, Under review).

#### Recruitment and screening data

During recruitment and screening, data were collected by the research team to identify how participants heard about the study to assess the marketing strategy. Some parent carers who opted not to take part provided the reasons for their decision, and these reasons were coded without any personal details.

#### Facilitator delivery checklists and session recordings

Self-report checklists, completed jointly by Lead and Assistant Facilitators after each session, were used to record the content that had been covered (adherence), the duration of sessions (dose), how well the facilitator felt the session went, and the participants’ engagement. Facilitators also recorded participants’ attendance at each session, including reasons for non-attendance where provided by participants.

Group sessions were audio-recorded and nine modules were randomly sampled to assess fidelity of content delivery, using the same checklist as used by facilitators. The nine module recordings were assessed using the checklist by one researcher (BCF) and independently double-scored by a second researcher (AB). Checklist scores were compared between both researchers, and between the researchers and the facilitators to assess the proportion of agreement in identifying delivered content.

#### Participant feedback forms and interviews

Feedback forms were completed by participants in both arms about the programme content, delivery, their experiences, and any contamination between trial arms.

Twelve participants in the intervention arm who attended group sessions (two from each site) and six participants from the control arm (one from each site) were interviewed about their experience and perceived impact of the programme, and about acceptability of trial processes. Four participants in the intervention arm who did not attend any group sessions were interviewed to ask about barriers to attendance.

### Qualitative analyses

Transcribed participant interviews and free-text data from participant questionnaires (feedback forms) were uploaded to NVivo (version 12). A coding framework was developed by three researchers (BCF, AB, and JL) to categorise and analyse the data. Interview transcripts (*n* = 18) were coded by BCF, with 9 (41%) double-coded by AB and JL. Interviews with non-attenders (*n* = 4) were summarised. Qualitative data from screening and attendance records were recorded in Excel spreadsheets and coded by BCF and checked by GB, with final codes agreed by both.

### Quantitative analyses

Descriptive statistics were used to summarise recruitment and retention of participants, demographics, attendance, and fidelity of intervention delivery, as well as participants’ feedback on the acceptability of the trial design.

Outcomes were compared between the trial arms at the post-intervention and follow-up data collection points according to the arm that participants were randomised to in keeping with the intention-to-treat principle. Random effects (“multilevel”) linear regression models were fitted to compare continuous outcomes, allowing for clustering within groups in the intervention arm of the trial. Satterthwaite’s degrees of freedom correction was used [43], given the small number of groups (clusters). Unadjusted analyses and analyses adjusted for study site were carried out. Missing data were not imputed. These analyses were exploratory, and *p* values are not reported in line with the extension to the CONSORT statement for reporting randomised pilot and feasibility studies [44]. We report estimates of correlations between baseline and follow-up for the outcome measures as well as estimates of standard deviations for the outcomes, with 95% confidence intervals.

## Results

### Feasibility of trial design

#### Participants

One hundred and ninety-three parent carers enquired about taking part in the study. Of those, 143 (74.1% (95% confidence interval: 67.3 to 80.1%)) were formally assessed for eligibility, with the remaining 50 not responding for further contact after initial enquiries. Of those who enquired about the study, 141 indicated how they heard about the study (Table 1).

**Table 1:** Frequency of recruitment sources

Source of study information	Number (%)
Social media	41 (21.2)
Event attended/held by study team	25 (13.0)
School letter or school staff contact	16 (8.3)
Parent Carer group	16 (8.3)
Friend/word of mouth	11 (5.7)
News story	11 (5.7)
Study advert shared by an organisation	8 (4.1)
PenCRU Family Faculty	5 (2.6)
Facilitator advert	3 (1.6)
Poster/flyer	3 (1.6)
Children’s services	1 (0.5)
Other email	1 (0.5)
No information provided	52 (26.9)
Total	193

Ninety-two participants (47.7% of those who expressed an interest (95% confidence interval: 40.4% to 55.0%)) provided written informed consent in person with a researcher before completing measures and were randomised (intervention *n* = 47, control *n* = 45). A minimum of 8 participants was required for each site in order to ensure that at least four parent carers would be allocated to attend group sessions, but the target was to recruit 16 per site. The numbers recruited varied by site with two sites recruiting under target (Torquay *n* = 13; Minehead *n* = 12) and one site recruiting over target (Plymouth *n* = 20). Recruitment improved over time, meeting our target in the last three sites. Of the two sites that were under target, one was offering evening sessions (the 12 × 2-h session format) and the other was in a geographically remote coastal town. Fifty parent carers opted not to take part in the study; the reasons that they gave are presented in Table 2. One participant in the Plymouth site withdrew from the study after providing written consent, but before randomisation. One participant in the control arm (Torquay site) withdrew from the study after allocation.

**Table 2:** Reasons for not consenting to take part

Reason	Number (%)
Distance/travel to group delivery site	18 (36.0)
Childcare	10 (20.0)
Time of group sessions not suitable	8 (16.0)
Work	7 (14.0)
Missed recruitment deadline	4 (8.0)
Parent health	1 (2.0)
Babe in arms	1 (2.0)
Pregnant	1 (2.0)
Total	50

Participants were aged 42.5 (8.0) years (mean (SD)), 96% female and 97% white. Sixty-three percent of participants were married, 45% were employed either part-time or full-time, and 16% lived in postcodes ranked in the most deprived quintile based on the Index of Multiple Deprivation 2019 [45]. Descriptive statistics of participants’ demographic characteristics at baseline are presented in Table 3. Baseline scores on outcome measures by trial arm status are presented in Table 4. Characteristics were broadly similar between arms, with the exception of the number of participants with at least moderate depression, which was higher in the control arm (*n* = 30; 67%) than in the intervention arm (*n* = 18; 42%).

**Table 3:** Baseline demographics of study participants by trial arm status

Characteristic	Intervention (***N*** = 47)^**a**^	Control (***N*** = 45 )^**b**^	All (***N*** = 92)^**c**^
Site			
Torquay, n (%)	7 (15)	6 (13)	13 (14)
Plymouth, n (%)	10 (21)	9 (20)	19 (21)
Minehead, n (%)	6 (13)	6 (13)	12 (13)
Dawlish, n (%)	8 (17)	8 (18)	16 (17)
Bideford, n (%)	8 (17)	8 (18)	16 (17)
St Austell, n (%)	8 (17)	8 (18)	16 (17)
Female parent, n (%)	44 (94)	44 (98)	88 (96)
Age of parent, mean (SD)	42.2 (8.9)	42.8 (7.1)	42.5 (8.0)
Number of children			
One, n (%)	12 (26)	7 (16)	19 (21)
Two, n (%)	24 (51)	30 (67)	54 (59)
Three, n (%)	6 (13)	5 (11)	11 (12)
Four, n (%)	5 (11)	2 (4)	7 (8)
Five, n (%)	0 (0)	1 (2)	1 (1)
Female index child ^d^, n (%)	10 (28)	10 (27)	20 (27)
Age of index child ^d^, mean (SD)	11.4 (5.0)	11.3 (5.1)	11.3 (5.0)
Relationship status			
Married, n (%)	26 (55)	32 (71)	58 (63)
Civil partnership, n (%)	2 (4)	0 (0)	2 (2)
Single, n (%)	7 (15)	4 (9)	11 (12)
Divorced, n (%)	9 (19)	6 (13)	15 (16)
Separated, n (%)	2 (4)	3 (7)	5 (5)
Widowed, n (%)	1 (2)	0 (0)	1 (1)
Ethnicity			
White, n (%)	45 (96)	44 (98)	89 (97)
Mixed multiple, n (%)	1 (2)	1 (2)	2 (2)
Other, n (%)	1 (2)	0 (0)	1 (1)
Employment status			
Full-time, n (%)	2 (4)	3 (7)	5 (6)
Full-time self-employed, n (%)	0 (0)	1 (2)	1 (1)
Part-time, n (%)	11 (24)	13 (30)	24 (27)
Part-time self-employed, n (%)	6 (13)	4 (9)	10 (11)
Parent carer, n (%)	23 (50)	21 (49)	44 (49)
Unemployed, n (%)	4 (9)	0 (0)	4 (4)
Full time student, n (%)	0 (0)	1 (2)	1 (1)
Educational qualification			
1 to 4 GCSEs/equivalent, n (%)	4 (9)	3 (7)	7 (8)
5+ GCSEs/equivalent, n (%)	8 (18)	5 (12)	13 (15)
2+ A levels, n (%)	6 (13)	6 (14)	12 (14)
Degree, n (%)	20 (44)	21 (49)	41 (47)
Other, n (%)	7 (16)	8 (19)	15 (17)
Total household income per week			
Up to £150, n (%)	4 (9)	0 (0)	4 (5)
£151 to £200, n (%)	1 (2)	2 (4)	3 (3)
£201 to £250, n (%)	9 (21)	6 (13)	15 (17)
£251 to £300, n (%)	2 (7)	4 (9)	6 (7)
£301 to £350, n (%)	7 (16)	9 (20)	16 (18)
£351 or above, n (%)	20 (47)	24 (53)	44 (50)
Housing tenure			
Owned outright, n (%)	6 (13)	3 (7)	9 (10)
Shared ownership, n (%)	0 (0)	1 (2)	1 (1)
Mortgage/loan, n (%)	16 (34)	26 (59)	42 (46)
Privately rented, n (%)	10 (21)	5 (11)	15 (16)
Council rented, n (%)	8 (17)	4 (9)	12 (13)
Other social rented, n (%)	6 (13)	5 (11)	11 (12)
Other, n (%)	1 (2)	0 (0)	1 (1)
IMD deprivation quintile-based group			
Most deprived, n (%)	8 (17)	7 (16)	15 (16)
2nd group, n (%)	16 (34)	11 (24)	27 (29)
3rd group, n (%)	10 (21)	15 (33)	25 (27)
4th group, n (%)	9 (19)	10 (22)	19 (21)
Least deprived	4 (9)	2 (4)	6 (7)
AMC concern score (index child) d, mean (SD)	11.3 (3.5)	12.9 (3.8)	12.0 (3.7)
AMC impact score (index child) d, mean (SD)	35.0 (13.3)	40.2 (14.3)	37.5 (14.0)

^a^ Sample size for intervention arm ranges from 36 to 47.

^b^ Sample size for control arm ranges from 32 to 45.

^c^ Total sample size ranges from 73 to 92.

*SD* standard deviation, *n* numerator, *N* denominator (sample size)

**Table 4:** Baseline scores on outcome measures by trial arm status

Characteristic	Intervention (***N*** = 47)^**a**^	Control (***N*** = 45)^**b**^	All (***N*** = 92)^**c**^
WEMWEBS wellbeing score, mean (SD)	40.2 (7.7)	38.1 (5.8)	39.2 (6.9)
PHQ-9 depression scale score, mean (SD)	10.2 (5.7)	11.5 (4.9)	10.9 (5.3)
At least moderate depression (PHQ-9 score ≥10)	18 (42)	30 (67)	48 (55)
Parent assessment of protective factors			
Total score, mean (SD)	3.8 (0.6)	3.5 (0.5)	3.7 (0.5)
Parental resilience, mean (SD)	3.8 (0.7)	3.7 (0.6)	3.7 (0.6)
Social connections, mean (SD)	3.7 (0.9)	3.1 (1.0)	3.4 (1.0)
Concrete support, mean (SD)	3.8 (0.7)	3.6 (0.7)	3.7 (0.7)
Social/emotional competence, mean (SD)	4.0 (0.6)	3.9 (0.6)	3.9 (0.6)
Health-promoting activities, mean (SD)	25.0 (9.6)	25.8 (8.2)	25.4 (8.9)
Patient activation measure, mean (SD)	53.7 (12.1)	53.9 (10.8)	53.8 (11.4)
EQ-5D-5L—mobility			
No problems, n (%)	37 (79)	30 (68)	67 (74)
Slight problems, n (%)	2 (4)	13 (30)	15 (16)
Moderate problems, n (%)	5 (11)	1 (2)	6 (7)
Severe problems, n (%)	2 (4)	0 (0)	2 (2)
Extreme problems, n (%)	1 (2)	0 (0)	1 (1)
EQ-5D-5L—self-care			
No problems, n (%)	36 (77)	42 (95)	78 (86)
Slight problems, n (%)	7 (15)	2 (5)	9 (10)
Moderate problems, n (%)	4 (9)	0 (0)	4 (4)
Severe problems, n (%)	0 (0)	0 (0)	0 (0)
Extreme problems, n (%)	0 (0)	0 (0)	0 (0)
EQ-5D-5L—usual activities			
No problems, n (%)	20 (43)	18 (40)	38 (41)
Slight problems, n (%)	18 (38)	16 (36)	34 (37)
Moderate problems, n (%)	4 (9)	10 (22)	14 (15)
Severe problems, n (%)	3 (6)	1 (2)	4 (4)
Extreme problems, n (%)	2 (4)	0 (0)	2 (2)
EQ-5D-5L—pain/discomfort			
No problems, n (%)	14 (30)	14 (31)	28 (31)
Slight problems, n (%)	17 (37)	23 (51)	40 (44)
Moderate problems, n (%)	11 (24)	6 (13)	17 (19)
Severe problems, n (%)	3 (7)	2 (4)	5 (5)
Extreme problems, n (%)	1 (2)	0 (0)	1 (1)
EQ-5D-5L—anxiety/depression			
No problems, n (%)	7 (15)	6 (14)	13 (14)
Slight problems, n (%)	18 (39)	19 (43)	37 (41)
Moderate problems, n (%)	14 (30)	15 (34)	29 (32)
Severe problems, n (%)	5 (11)	4 (9)	9 (10)
Extreme problems, n (%)	2 (4)	0 (0)	2 (2)
EQ-5D-5L—visual analogue scale, mean (SD)	58.9 (22.4)	56.3 (22.5)	57.7 (22.3)
EQ-5D-5L—health state utility value, mean (SD)	0.670 (0.244)	0.723 (0.203)	0.696 (0.225)
ICECAP-A—feeling settled and secure			
Most negative category, n (%)	5 (11)	5 (11)	10 (11)
2nd category, n (%)	21 (45)	25 (56)	46 (50)
3rd category, n (%)	19 (40)	15 (33)	34 (37)
Most positive category, n (%)	2 (4)	0 (0)	2 (2)
ICECAP-A—love, friendship and support			
Most negative category, n (%)	2 (4)	0 (0)	2 (2)
2nd category, n (%)	17 (37)	21 (48)	38 (42)
3rd category, n (%)	21 (46)	19 (43)	40 (44)
Most positive category, n (%)	6 (13)	4 (9)	10 (11)
ICECAP-A—being independent			
Most negative category, n (%)	0 (0)	0 (0)	0 (0)
2nd category, n (%)	4 (9)	8 (18)	12 (14)
3rd category, n (%)	27 (61)	21 (48)	48 (55)
Most positive category, n (%)	13 (30)	15 (34)	28 (32)
ICECAP-A—achievement and progress			
Most negative category, n (%)	2 (4)	2 (5)	4 (4)
2nd category, n (%)	23 (51)	22 (50)	45 (51)
3rd category, n (%)	19 (42)	17 (39)	36 (40)
Most positive category, n (%)	1 (2)	3 (7)	4 (4)
ICECAP-A—enjoyment and pleasure			
Most negative category, n (%)	1 (2)	0 (0)	1 (1)
2nd category, n (%)	26 (58)	32 (73)	58 (65)
3rd category, n (%)	18 (40)	12 (27)	30 (34)
Most positive category, n (%)	0 (0)	0 (0)	0 (0)
ICECAP-A—tariff, mean (SD)	0.674 (0.157)	0.637 (0.154)	0.655 (0.156)

^a^ Sample size for intervention arm ranges from 36 to 47

^b^ Sample size for control arm ranges from 32 to 45

^c^ Total sample size ranges from 73 to 92

^d^ “Index child” is the first child for whom the parent reported data at baseline

*SD* standard deviation, *n* numerator, *N* denominator (sample size)

#### Acceptability of trial processes

Interview participants were mostly satisfied with the delivery format they were allocated to, with more positive views (from both intervention and control participants) expressed about the group programme (Table 5). Participants also discussed different circumstances and preferences for delivery formats, which could be better accounted for if participants were given a choice of joining the programme in-person or online.

**Table 5:** Example quotes from participant interviews about acceptability of trial processes

*Views about allocation* I would have been happy with either way, but I was really pleased to have been assigned to the group for the reason that I was hoping to meet other people who are in a similar situation to me... [Intervention participant] I was a bit disappointed because I thought I could do this every week, it will get me out of the house, because that would have given me the incentive to go somewhere and it would have been a walk and some exercise to get there. [Control participant] …depending on your personal circumstances, work, things like that… I know it’s supposed to be a random choice about who did the meetings or who did it online, but maybe a slight conversation with some parent carers to say ‘Which would be better for you?’ then maybe a choice rather than it being too random might be better. [Intervention participant] *Views about measures* I don’t really mind doing questionnaires anyway, depending on how it’s written, but they weren’t onerous questionnaires, they were alright… I just did them and was pleased with the vouchers. I enjoyed those actually, I thought they were good. It was just nice to be asked the right questions for once. I found it really emotional at the beginning and I must admit, I would have preferred to have done it on my own because I got really choked up at times. (…) It was at a time when I was really struggling emotionally. I really didn’t want to be observed. (…) The end questionnaire, I felt slightly nervous because I didn’t remember what I put in the first one. I thought, I really want this to be a success because I think it is great, but if I don’t answer the right questions will it not be a success? I had no problems with [completing the questionnaires] because that is just something that we are used to doing now, to be honest. There was a fear, I will not lie, at the back of my mind of safeguarding issues. It always… constantly, as a parent of a disabled child, I think it is always… you worry that you fill in a questionnaire and somebody… a red flag is going to go up and somebody is going to come down on you and say, ‘Look, we have got concerns for your children’s safety’… I was a little bit anxious about filling it out but I did answer it honestly. Ironically… I actually think my after ones are possibly the answers that are not quite so good in terms of my mental health than the beginning ones but like I say, that is circumstances out of your control because it is nothing to do with reflecting on the course. It is just purely that we have had so much negativity and bad things happening in the last six weeks that that is possibly reflected in that.	

Interviewees found the questionnaires (and the time to complete them) acceptable, and the questions asked relevant. Although the vast majority of interviewees had very positive views about the programme, a few were concerned about whether their positive experience was captured by the questionnaires. Three interviewees described how honestly answering the questionnaires made them concerned about triggering safeguarding processes. They also found it easy to complete the questionnaires online (especially when possible to do it on their phone), whereas found completing the initial questionnaire in-person more uncomfortable and pressured.

#### Contamination between trial arms

Four participants in the intervention arm (9%) and four participants in the control arm (9%) reported discussing the programme with participants in the other arm. No further details were collected about possible contamination.

#### Attrition

Outcome data were collected from 91% (95% confidence interval 84% to 96%) of participants at post-intervention (98% intervention arm; 84% control arm) and 90% (95% confidence interval 82% to 95%) of participants at 6-month follow-up (98% intervention arm; 82% control arm). However, the number of participants that provided complete data for scoring and analysis of outcomes ranged from 74 (80%) to 83 (90%) at post-intervention and from 71 (77%) to 78 (85%) at 6-month follow-up. There was no evidence that follow-up status was related to outcome scores at baseline.

#### Outcomes

Table 6 presents the mean differences between trial arms in outcomes at the first follow-up (post-intervention) and the second follow-up (6 months post-intervention). At the first follow-up, an adjusted mean difference of 3.2 (95% confidence interval − 0.8 to 7.3) was found for the primary outcome WEMWBS wellbeing measure, but this difference diminished by the second follow-up (adjusted mean difference − 0.7 (95% confidence interval −5.3 to 3.9).

**Table 6:** Comparison of outcomes between trial arms

Outcome	Intervention (I)		Control (C)		Mean Difference	Adjusted mean difference	
	N	mean (SD)	N	mean (SD)	Crude (I – C)	estimate	95% CI
*First follow-up*							
WEMWEBS wellbeing score	44	46.4 (7.0)	34	43.4 (6.3)	3.1	3.2	− 0.8 to 7.3
PHQ-9 depression scale score	42	7.8 (4.4)	35	8.8 (4.8)	− 1.0	− 0.9	− 3.7 to 1.9
Parent assessment of protective factors							
Total score	42	4.0 (0.5)	32	3.8 (0.5)	0.1	0.1	− 0.2 to 0.5
Parental resilience	45	4.1 (0.6)	35	3.9 (0.5)	0.2	0.2	− 0.2 to 0.5
Social connections	43	3.7 (1.0)	33	3.6 (1.0)	0.1	0.1	− 0.5 to 0.8
Concrete support	44	3.9 (0.6)	34	3.8 (0.6)	0.1	0.1	− 0.3 to 0.5
Social/emotional competence	44	4.2 (0.5)	33	4.0 (0.6)	0.2	0.2	− 0.2 to 0.5
Health-promoting activities	44	30.6 (8.7)	35	28.6 (7.7)	2.0	2.0	− 3.9 to 8.0
Patient activation measure	43	59.1 (13.2)	34	57.6 (10.1)	1.4	1.4	− 5.8 to 8.7
*Second follow-up*							
WEMWEBS wellbeing score	43	44.4 (8.3)	35	44.9 (6.7)	− 0.5	− 0.7	− 5.3 to 3.9
PHQ-9 depression scale score	42	9.1 (6.4)	31	8.5 (4.4)	0.8	0.9	− 3.1 to 4.8
Parent assessment of protective factors							
Total score	40	4.0 (0.5)	32	3.8 (0.5)	0.1	0.1	− 0.2 to 0.4
Parental resilience	45	4.0 (0.5)	36	4.0 (0.5)	0.006	0.002	− 0.3 to 0.3
Social connections	43	3.6 (1.0)	34	3.5 (1.1)	0.1	0.1	− 0.5 to 0.7
Concrete support	45	4.0 (0.7)	36	3.8 (0.7)	0.2	0.2	− 0.2 to 0.6
Social/emotional competence	43	4.2 (0.5)	35	4.0 (0.6)	0.1	0.1	− 0.3 to 0.5
Health-promoting activities	42	29.5 (9.7)	34	30.5 (9.8)	− 1.2	− 1.1	− 10.9 to 8.7
Patient activation measure	44	59.1 (15.2)	34	61.7 (12.7)	− 2.7	− 2.7	− 11.1 to 5.8

*SD* standard deviation, *CI* confidence interval, *N* denominator (sample size)

Twenty participants (21.7%) reported thoughts of self-harm or suicidal ideation on question 9 of the PHQ-9 at least once during the study (15 participants reported once; 5 participants reported twice). Of the 25 reports, 13 were at baseline, 3 at the first follow-up, and 9 at the second follow-up.

Correlations between baseline and follow-up scores on continuous outcomes were generally moderate (Table 7). Estimated standard deviations for the study outcomes are presented by trial arm status in Table 8.

**Table 7:** Pearson correlation (r) between baseline score and follow-up score for each outcome

Outcome	First follow-up		Second follow-up	
	Number	r (95% CI)	Number	r (95% CI)
WEMWEBS wellbeing	74	0.57 (0.39 to 0.71)	76	0.40 (0.19 to 0.57)
PHQ-9 depression	73	0.49 (0.29 to 0.65)	70	0.65 (0.48 to 0.76)
Parent assessment of protective factors^a^	61	0.78 (0.66 to 0.87)	62	0.68 (0.52 to 0.79)
Health-promoting activities	76	0.46 (0.26 to 0.62)	73	0.45 (0.25 to 0.62)
Parent activation measure	74	0.54 (0.35 to 0.68)	75	0.62 (0.46 to 0.74)

^a^ Parent assessment of protective factors

*r* Pearson correlation coefficient, *CI* confidence interval, *N* denominator (sample size)

**Table 8:** Estimated standard deviations (SDs) for study outcomes by trial arm status

Outcome	Intervention		Control		All	
	N	SD (95% CI)	N	SD (95% CI)	N	SD (95% CI)
*Baseline*						
WEMWEBS wellbeing					87	6.9 (6.0 to 8.1)
PHQ-9 depression					88	5.3 (4.6 to 6.2)
Parent assessment of protective factors ^a^					78	0.54 (0.46 to 0.64)
Health-promoting activities					88	8.9 (7.7 to 10.4)
Parent activation measure					88	11.4 (9.9 to 13.4)
*First follow-up*						
WEMWEBS wellbeing	44	7.0 (5.8 to 8.9)	34	6.3 (5.1 to 8.3)	78	6.8 (5.9 to 8.1)
PHQ-9 depression	42	4.4 (3.6 to 5.6)	35	4.8 (3.8 to 6.2)	77	4.6 (3.9 to 5.4)
Parent assessment of protective factors ^a^	42	0.53 (0.44 to 0.68)	32	0.45 (0.36 to 0.60)	74	0.50 (0.43 to 0.60)
Health-promoting activities	44	8.7 (7.2 to 11.1)	35	7.7 (6.2 to 10.1)	79	8.3 (7.2 to 9.8)
Parent activation measure	43	13.2 (10.9 to 16.8)	34	10.1 (8.1 to 13.3)	77	11.9 (10.3 to 14.1)
*Second follow-up*						
WEMWEBS wellbeing	43	8.3 (6.8 to 10.5)	35	6.7 (5.4 to 8.7)	78	7.5 (6.5 to 9.0)
PHQ depression	42	6.4 (5.2 to 8.1)	31	4.4 (3.5 to 5.9)	73	5.6 (4.8 to 6.7)
Parent assessment of protective factors ^a^	40	0.54 (0.44 to 0.69)	32	0.48 (0.38 to 0.63)	72	0.51 (0.44 to 0.61)
Health-promoting activities	42	9.7 (8.0 to 12.3)	34	9.8 (7.9 to 12.9)	76	9.7 (8.3 to 11.5)
Parent activation measure	44	15.2 (12.6 to 19.3)	34	12.7 (10.2 to 16.7)	78	14.2 (12.2 to 16.8)

^a^ Total score on Parent assessment of protective factors

*SD* standard deviation, *CI* confidence interval, *N* denominator (sample size)

### Feasibility of cost-effectiveness framework

#### Intervention resource use and costs

The resources required for the delivery of the programme were identified and measured, and unit costs obtained for all elements of resource use. These are detailed in Additional file 2.

#### Resource use questionnaire

A bespoke resource use questionnaire was developed in collaboration with parent carers. This involved two face-to-face meetings and further input and feedback via email and telephone. The designed measure comprised the core items for a standardized resource use measure [46], in addition to questions pertaining to other NHS, Social Services, and Local Authority support. Items were also included in relation to ‘Support from others’, ‘Own expenses’, ‘Services/resources for your child’, and ‘Other services/resources used or things done in the last 6 months that improved your health/wellbeing’. Responses to the questionnaire at 6 months post-intervention by trial arm are provided in Additional file 3.

#### QALY and WALY measures

Table 9 presents the EQ-5D-5L QALYs and ICECAP-A WALYs by trial arm. These figures indicate the completeness of the data at each of the baseline and follow-up points.

**Table 9:** EQ-5D-5L quality-adjusted life-years (QALYs) and ICECAP-A wellbeing-adjusted life years (WALYs), by trial arm

Measure: time point	Intervention (I)		Control (C)		Mean difference	Adjusted mean difference*	
	N	mean (SD) [range]	N	mean (SD) [range]	(I-C) (unadjusted)	estimate	95% CI
EQ-5D-5L: baseline	44	0.670 (0.244) [− 0.098 to 1]	44	0.723 (0.203) [− 0.239 to 1]			
EQ-5D: 14 weeks	45	0.659 (0.269) [− 0.225 to 1]	38	0.742 (0.166) [0.238 to 1]	− 0.083	− 0.099	− 0.203 to 0.006
EQ-5D: 9.5 months	40	0.645 (0.228) [− 0.032 to 1]	31	0.675 (0.185) [0.364 to 1]	− 0.030	− 0.029	− 0.130 to 0.072
EQ-5D QALYs: 9.5 months	35	0.488 (0.117) [0.195 to 0.644]	26	0.519 (0.090) [0.301 to 0.663]	− 0.031	− 0.024	− 0.079 to 0.030
ICECAP-A: baseline	44	0.674 (0.157) [0.291 to 0.920]	44	0.637 (0.154) [0.269 to 0.942]			
ICECAP-A: 14 weeks	45	0.719 (0.169) [0.363 to 0.969]	34	0.718 (0.138) [0.371 to 0.934]	0.001	− 0.013	− 0.072 to 0.047
ICECAP-A: 9.5 months	44	0.713 (0.189) [0.251 to 1]	33	0.707 (0.159) [0.432 to 1]	0.006	0	− 0.072 to 0.072
ICECAP-A WALYs: 9.5 months	40	0.512 (0.108) [0.239 to 0.663]	27	0.519 (0.083) [0.341 to 0.699]	− 0.007	− 0.007	− 0.044 to 0.029

*Adjusted for study site and baseline score of the outcome measure

### Feasibility of delivery

According to facilitators’ self-report checklists, 90% of activities were delivered across all groups. Scores from researcher checklists of the nine recorded modules similarly indicated that 91% of activities were delivered. The activities that were not delivered only included some icebreakers and concluding activities, not the main programme content. The two researchers were in agreement for 98% of assessed activities (only disagreeing in one concluding activity), showing high reliability of using the checklists to assess the content delivered.

#### Group session attendance

Sixteen (34%) participants allocated to the intervention arm did not attend any group intervention sessions. Twenty-seven of the 47 (57%, 95% confidence interval: 42% to 72%) participants in the intervention arm attended at least two-thirds of the sessions. The minimum number of participants attending any sessions was two, which occurred in six sessions across four groups.

There was no clear evidence of a relationship between attendance and baseline scores on outcome measures. Four of the participants who did not attend any sessions and were interviewed reported practical reasons for non-attendance, such as the distance to travel to sessions, child health, parent health, childcare, or work commitments. Twenty-two (47%) intervention arm participants also missed one or more sessions. The reasons provided for missing sessions were most commonly work commitments and parent or child health; frequencies are presented in Table 10.

**Table 10:** Reasons for missed sessions

Reason	Number (%)
Work	13 (18%)
Parent unwell	12 (16%)
Child unwell	11 (15%)
Unknown	8 (11%)
Other commitment	7 (10%)
Parent health appointment	5 (7%)
Child health appointment	5 (7%)
No further contact	5 (7%)
Childcare	2 (3%)
Bereavement	1 (1%)
Other family member unwell	1 (1%)
Missed communication	1 (1%)
Forgot session was running	1 (1%)
Job interview	1 (1%)
Total	73

#### Engagement with online resources

Participants reported their use of the online resources at first and second follow-up. A pattern emerged in which participants in the intervention arm were less likely to use the resources,and used fewer of them, compared to those in the control arm (Table 11).

**Table 11:** Self-reported use of online material in the intervention arm

Amount	By first follow-up		By second follow-up	
	Intervention	Control	Intervention	Control
	***N*** = 47	***N*** = 45	***N*** = 47	***N*** = 45
None, n (%)	20 (43%)	4 (9%)	27 (57%)	10 (22%)
25%, n (%)	13 (28%)	1 (2%)	11 (23%)	8 (18%)
50%, n (%)	3 (6%)	3 (7%)	5 (11%)	7 (16%)
More than 50%, n (%)	8 (17%)	20 (44%)	2 (4%)	12 (27%)
Missing, n (%)	3 (6%)	17 (38%)	2 (4%)	8 (18%)

*n* numerator, *N* denominator (sample size)

## Discussion

This study has demonstrated that it was feasible to set up and deliver the HPC programme in the community and the trial design and cost-effectiveness framework appeared generally feasible and acceptable to participants. Six venues were established for group delivery in a variety of community settings and in city and rural locations in Cornwall, Devon, and Somerset. Two Lead Facilitators, six Assistant Facilitators, and three reserve facilitators were recruited and trained to deliver the programme. According to facilitators’ self-reports and a sample of researcher-coded session recordings, the group-based programme was delivered as designed across all groups.

There was considerable interest in the study from parent carers, indicating that a similar recruitment strategy could be effective for recruiting participants to sites in a definitive trial. Social media and events were found to be the most helpful elements of the recruitment strategy and should be utilised in any subsequent evaluation or implementation. A sufficient number of participants were recruited in each study site and randomised to form viable groups in the intervention arm (*n* = 92).

Attrition of participants from the trial was low, with outcome data collected from 91% (95% confidence interval 84% to 96%) of participants at post-intervention and 90% (95% confidence interval 82% to 95%) of participants at 6-month follow-up. This was above the study target of 80% and was achieved utilising a combination of automated email reminders, phone calls, text messages and vouchers as acknowledgements. This system should be replicated in a definitive trial to promote similar follow-up rates. However, given the findings in a review of attrition in external pilot trials, which found a large amount of variability in the difference in attrition rates between pilot trials and their associated full trials, any estimate of likely follow-up should still be cautious [47].

In addition, the number of participants that provided completed questionnaires with no missing data on outcome measures ranged from 74 (80%) to 83 (90%) at post-intervention and from 71 (77%) to 78 (85%) at 6-month follow-up. Generally, across all measures, the earlier items were more likely to be completed than the later items. On inspection of the data and electronic patient-reported outcomes system, it appears that this may have been in part due to the layout and need for scrolling to later items on some devices. The formatting and presentation of the measures on all types of devices should be reviewed in a subsequent study to prevent this. The length and number of questionnaires should also be considered as fatigue may have been a factor.

The trial design was generally acceptable to parent carers, although many would have preferred to choose their way of accessing the programme rather than undergo random allocation. A partially randomised patient preference trial could be considered to increase participation in a future trial [48]. The type of control used in future could also be considered and discussed with patient and public involvement group members to see if there might be a more acceptable option, such as a possible waitlist control. The outcome measures were largely acceptable to participants, although some technical problems with the electronic patient-reported outcomes system were reported. The electronic patient-reported outcomes system should be revisited and tested by users before implementation in the definitive trial.

The identification of resources required to deliver the intervention and their associated costs will be used to plan and budget for future implementation, including for a definitive trial. As the programme is refined and implementation by community organisations is planned, the cost implications of any changes will be explored. The cost of implementing the programme is highly likely to reduce over time as facilitators are trained to deliver repeated groups and certain materials can be reused.

The number recruited to take part represented only 50% of those who initially enquired, and the most common reasons for not taking part were practical barriers to attending groups. Among those taking part, attendance at group sessions was variable and often lower than intended. Further exploration of ways to reduce barriers to access and participation, which may include reducing the time from recruitment to randomisation and the feasibility of offering further support, such as providing funded childcare, is needed. Larger minimum group sizes at the outset would help to ensure that the attendance is sufficient to enable the programme to be delivered with fidelity to function (see findings from the process evaluation reported separately (Lloyd J, Bjornstad G, Borek A, Cuffe-Fuller B, Fredlund M, McDonald A, Tarrant M, Berry V, Wilkinson K, Mitchell S, et al: The Healthy Parent Carers programme: mixed-methods process evaluation and refinement of a health promotion intervention, Under review)).

This study provides important information about parameters that will be used for calculating the sample size for the definitive trial, including the variation in group sizes for intervention delivery, the follow-up percentage and the standard deviation of the putative primary outcome (the WEMWBS).

This study was not conducted in an ethnically diverse part of the UK and as such does not provide information about the relevance and acceptability of the programme to parent carers from a range of ethnical and cultural backgrounds, nor did we seek to establish the feasibility of delivering group sessions with interpreters for those who cannot communicate in English. Most work on ethnicity and parenting interventions has been conducted in the USA and is equivocal about whether outcomes differ by ethnicity [49, 50]. Understanding how ethnicity might influence the development of a shared group identity, recognised as a key mechanism of action for group-based programmes, will be explored in future work.

Given the current context of the Covid-19 pandemic and need for social distancing, delivering in-person groups is not possible and will likely be affected in the near future. Therefore, we will explore virtual groups using online videoconferencing as an alternative mode of delivery.

## Conclusions

The study demonstrated that it was feasible to train facilitators and deliver the peer-led, group-based programme in community settings. The number of parent carers who expressed interest signifies the need for such a programme and the feasibility of recruiting to a definitive trial. Loss to follow-up was low. However, many interested parents were unable to take part and attendance was variable. Further research is needed to explore ways to reduce barriers to participation in person. Research assessing the feasibility and acceptability of programme content and delivery for more ethnically diverse groups, and potentially using interpreters is also needed to increase potential reach. Given the Covid-19 pandemic and delivery format feedback, there is also a need to investigate remote or blended delivery strategies. Although the results indicate that a definitive trial is feasible, programme impact would be strengthened through exploration of these uncertainties.

## Supplementary Information

**Additional file 1.** CONSORT 2010 checklist.

**Additional file 2.** Healthy Parent Carers Programme resource requirements and unit costs.

**Additional file 3.** Resource use at six months post-HPC intervention, by trial group.

## Data Availability

The datasets analysed during the current study are available from the corresponding author on reasonable request via Open Research Exeter:  10.24378/exe.3423.
