# Supplementary material for: Healthy Parent Carers: feasibility randomised controlled trial of a peer-led group-based health promotion intervention for parent carers of disabled children
Source: Pilot Feasibility Stud. 2021 Jul 23;7:144. doi: 10.1186/s40814-021-00881-5 (PMC8298691; doi:10.1186/s40814-021-00881-5)
Supplement: Supplementary file 2 — Additional file 2. Healthy Parent Carers Programme resource requirements and unit costs. [file 40814_2021_881_MOESM2_ESM.pdf]

Additional File 2: Healthy Parent Carers Programme resource requirements and unit costs

Resource item	*Quantity*	*Unit cost*	*Unit cost source*
**Intervention contact time:**	12 modules<br>x 2 hours per module<br>x 6 sites<br>= 144 hours		
Lead facilitators	1 Lead facilitator per module<br>= 144 hours	£50 per hour	Council for Disabled Children (CDC) trainer rate
Assistant facilitators	1 Assistant facilitator per module<br>= 144 hours	£14.71 per hour	Agenda for Change Band 5, Point 23
**Intervention non-contact time:**			
Lead facilitator preparation prior to module	12 modules<br>x approximately 1 hour preparation<br>x 6 sites<br>= approx. 72 hours	£50 per hour	CDC trainer rate
Lead and Assistant facilitator pre- and de-brief	12 modules<br>x approximately 30 minutes<br>x 6 sites<br>= approximately 36 hours	£50 per hour<br>+ £14.71 per hour	CDC trainer rate<br>Agenda for Change Band 5, Point 23
Additional contact with supervisor<br>(Lead and Assistant Facilitator)	7 contacts<br>x approximately 1 hour<br>= approximately 7 hours	£100 per hour<br>+ £50 per hour<br>+ £14.71 per hour	Intervention providers<br>CDC trainer rate<br>Agenda for Change Band 5, Point 23
**Equipment and materials:**	6 (i.e. per site)	£35	Intervention providers
Facilitator manual	8	£10	
Trolley	2	£15	
Speakers	2	£16	
Laptops	2	Facilitator owned	
**Room/venue hire**	1 site	5 venues - charities	Intervention providers
**Refreshments**	6 (sites)	£12	Intervention providers
**Travel:**			
Lead facilitators	6 (sites)	£245.70	Intervention providers
Assistant facilitators	6 (sites)	£147.60	

**Supervision:**			
Lead facilitators	2 facilitators x 6 hours<br>= 12 hours	£100 per hour<br>+ £50 per hour	Intervention providers<br>CDC trainer rate
Assistant facilitators	6 facilitators x 6 hours<br>= 36 hours	£100 per hour<br>+ £14.71 per hour	Intervention providers<br>Agenda for Change Band 5, Point 23
**Training:**			
Staffing:			
Supervisor training Lead facilitators	3 days (i.e. 7 hours per day)	£100 per hour	Intervention providers
Lead facilitator training	2 facilitators x 2 days<br>= 28 hours	£50 per hour	CDC trainer rate
Lead facilitator refresher training	2 facilitators x 1 day<br>= 14 hours	£50 per hour	CDC trainer rate
Lead facilitator providing Assistant facilitator training	2 facilitators x 5 days<br>= 70 hours	£50 per hour	CDC trainer rate
Assistant facilitator training	6 facilitators x 2 days<br>= 84 hours	£14.71 per hour	Agenda for Change Band 5, Point 23
Lead providing Assistant facilitator refresher training	2 facilitators x 1 day<br>= 14 hours	£50 per hour	CDC trainer rate
Assistant facilitator refresher training	6 facilitators x 1 day<br>= 42 hours	£14.71 per hour	Agenda for Change Band 5, Point 23
Refreshments:			
Lead facilitator training			Intervention providers
Assistant facilitator training			
Refresher training			
Travel:			
Lead facilitator training	1 supervisor + 2 facilitators	£50 per person	Intervention providers
Assistant facilitator training (incl. overnight stay for 3)	8 facilitators	£80 per person	
Refresher training	1 supervisor + 8 facilitators	£24 per person	
Equipment and materials:			Intervention providers
Training manual	3 (1 per trainer)	£10	
