# Supplementary material for: Healthy Parent Carers: feasibility randomised controlled trial of a peer-led group-based health promotion intervention for parent carers of disabled children
Source: Pilot Feasibility Stud. 2021 Jul 23;7:144. doi: 10.1186/s40814-021-00881-5 (PMC8298691; doi:10.1186/s40814-021-00881-5)
Supplement: Supplementary file 3 — Additional file 3. Resource use at six months post-HPC intervention, by trial group. [file 40814_2021_881_MOESM3_ESM.pdf]

Additional File 3: Resource use at six months post-HPC intervention, by trial group

Resource item	***Intervention* (n=45)**		***Control* (n=37)**	
	**n (%) used service**	**Contacts, group mean (SD) [range]**	**n (%) used service**	**Contacts, group mean (SD) [range]**
Primary care NHS services				
GP at surgery	30 (66.7)	1.9 (2.4) [1 to 12]	26 (70.3)	1.5 (1.5) [0 to 5]
GP at home	2 (4.8)	0.02 (0.2) [0 to 1]	0	0
GP via telephone	16 (36.4)	0.9 (1.6) [0 to 7]	16 (45.7)	0.9 (1.5) [0 to 5]
Physiotherapist at surgery	9 (20.5)	0.2 (0.6) [0 to 3]	0	0
Physiotherapist at home	0	0	1 (3.1)	0.4 (2.5) [0 to 14]
Occupational therapist at surgery	0	0	0	0
Occupational therapist at home	1 (2.3)	0.05 (0.3) [0 to 2]	2 (6.3)	0.03 (0.2) [0 to 1]
Practice nurse at surgery	23 (52.3)	0.9 (1.3) [0 to 5]	13 (41.9)	1 (1.9) [0 to 8]
Community nurse	0	0	0	0
Community mental health team	3 (6.8)	0.2 (0.9) [0 to 6]	2 (6.5)	0.6 (2.4) [0 to 10]
Community psychiatric nurse	0	0	0	0
Counsellor	7 (16.7)	1.6 (5.6) [0 to 30]	5 (17.2)	1.4 (4.7) [0 to 24]
Dietician/Nutritionist	0	0	1 (3.3)	0.07 (0.4) [0 to 2]
Other primary care services:				
Social Prescribing Service	2		0	
Outlook South West	1		0	
Weight management referral	1		0	
Slimming World	0		1	
Health problems related to weight	1		0	
Genetic Consultant appointment	1		0	
Hospital-based NHS services	**n (%) used service**	**Contacts, group mean (SD) [range]**	**n (%) used service**	**Contacts, group mean (SD) [range]**
Physiotherapist	9 (20.0)	0.4 (1.1) [0 to 6]	2 (5.4)	0.06 (0.3) [0 to 2]
Occupational therapist	0	0	0	0
Psychologist	2 (4.6)	0.05 (0.21) [0 to 1]	0	0
Psychiatrist	2	0.05 (0.21)	0	0

	(4.6)	[0 to 1]		
Pain clinic	4 (9.1)	0.1 (0.3) 0.2 [0 to 1]	0	0
Other hospital-based services:<br>Podiatry<br>Sleep clinic<br>Colonoscopy<br>Dental Centre<br>Ehlers-Danlos appointment<br>Endoscopy<br>Trauma surgery team	 0<br>0<br>1<br>1<br>1<br>1<br>1		 1<br>1<br>0<br>0<br>0<br>0<br>0	
Hospital stays/visits	**n (%) used service**	**Group mean (SD) [range]**	**n (%) used service**	**Group mean (SD) [range]**
Overnight stay in hospital	3 (7.3)	0.1 (0.4) [0 to 2]	1 (2.9)	0.03 (0.2) [0 to 1]
Day visit to hospital	3 (7.5)	0.1 (0.4) [0 to 2]	3 (9.4)	0.1 (0.4) [0 to 2]
A&E visit	9 (22.0)	0.4 (0.8) [0 to 3]	3 (9.4)	0.1 (0.3) [0 to 1]
Other NHS, Social Services and Local Authority support	**n (%) used service**	**Group mean (SD) [range]**	**n (%) used service**	**Group mean (SD) [range]**
Carers' Assessment<br><br>Carers' Assessment support received:<br>CAMHS Carer Support Worker<br>Peer support sessions<br>Carer's grant<br>Respite care for child<br>Meeting with Devon Carers<br>Carers' letter confirming entitled to payments<br>Carers' course<br>Healthy living gym membership<br>Meditation and relaxation course<br>Waiting to hear<br>No support given	8<br>(18.2)<br><br>0<br>0<br>2<br>1<br>1<br>1<br>1<br>1<br>1<br>0<br>0		4<br>(10.8)<br><br>1<br>1<br>0<br>0<br>0<br>0<br>0<br>0<br>0<br>1<br>1	
Social worker/Care manager appointments	4 (9.5)	0.3 (0.9) [0 to 4]	2 (5.4)	0.1 (0.2) [0 to 1]
Support via child's school<br><br>Support received via child's school:<br>Parent Support Advisor<br>Family Support Worker<br>Safe Families for Children support in home<br>PFSA support<br>School nurse<br>Timid to Tiger course	5<br>(11.9)<br><br>1<br>1<br>0<br>0<br>1<br>1		4<br>(10.8)<br><br>1<br>1<br>1<br>1<br>0<br>0	
Parent Carers support group sessions, last 6 months	12 (26.7)	4.7 (15.8) [0 to 100]	16 (43.2)	2.9 (5.1) [0 to 24]

Parent Carers support group sessions, average length		2.5 hours		1.9 hours
Parent Carers support groups sessions' aim:<br>Peer support<br>Family Support group<br>Psychoeducation group<br>Child play<br>Member of Parent Carers Council Cornwall Strategic Team<br>Help others<br>New skills<br>Christmas crafts	 12<br>0<br>1<br>0<br>0<br>0<br>0<br>1		 11<br>1<br>0<br>1<br>1<br>1<br>1<br>0	
Parent Carers support group name:<br>AIMS<br>CAMHS Carers group<br>Face to Face<br>Jumpstart/CAOT group<br>Minehead hub<br>NAS Exeter Parent Carers<br>North Devon Forum for Autism & ADHD<br>Tissues and Issues<br>Jungle Jacks quiet session<br>Affinity support group<br>Braunton Parent Carers support group<br>Breathe<br>In Touch<br>Parent Carer Council<br>Parent Carers Cornwall<br>Side by Side	 0<br>0<br>1<br>0<br>0<br>0<br>1<br>0<br>0<br>1<br>1<br>1<br>1<br>1<br>1<br>3		 1<br>1<br>1<br>1<br>1<br>1<br>3<br>2<br>1<br>0<br>0<br>0<br>0<br>0<br>0<br>0	
Carers support group sessions, last 6 months	5 (11.9)	3.2 (15.9) [0 to 100]	4 (11.4)	0.8 (3.5) [0 to 20]
Carers support group sessions, average length		1.75 hours		2 hours
Carers support groups sessions' aim:<br>Support<br>Peer support<br>Peer support (not autism specific)<br>Social time<br>Relaxation/Meditation<br>Gym<br>Crafts<br>To get out of house	 2<br>0<br>0<br>0<br>1<br>1<br>0<br>0		 0<br>2<br>1<br>1<br>1<br>0<br>1<br>1	
Carers support group name:<br>Devon Carers peer support<br>North Devon Forum for Autism & ADHD<br>Parent Carers Support group<br>Talking Café Carers group<br>Carers Torbay<br>Side by Side	 0<br>0<br>0<br>0<br>1<br>1		 1<br>1<br>1<br>1<br>0<br>0	
Free fitness groups	1	0.07 (0.5) [0 to 3]	0	0
Support from others	**n (%)**	**Group mean (SD)**	**n (%)**	**Group mean (SD)**

		[range]		[range]
Friends/family helped with tasks at home:	19 (42.2)		13 (35.1)	
Childcare,<br>average hours help per month		5.6 (10.4)<br>[0 to 40]		2.6 (6.0)<br>[0 to 25]
Housework/laundry,<br>average hours help per month		1.3 (3.5)<br>[0 to 14]		0.3 (1.1)<br>[0 to 6]
DIY/home maintenance,<br>average hours help per month		0.5 (1.8)<br>[0 to 10]		0.4 (1.3)<br>[0 to 6]
Gardening,<br>average hours help per month		0.3 (1.0)<br>[0 to 5]		0.2 (0.5)<br>[0 to 2]
Shopping,<br>average hours help per month		0.5 (1.8)<br>[0 to 9]		0.7 (2.0)<br>[0 to 10]
Friends/relatives stayed off work to help	2 (4.8)		1 (2.7)	
Online support groups,<br>average hours per week	10	2.7 (15.6)<br>[0 to 100]	10	1.3 (2.8)<br>[0 to 10]
Own expenses	**n (%)**	**Group mean (SD) [range]**	**n (%)**	**Group mean (SD) [range]**
Personally paid for help with tasks, therapies, services or activities for own health/wellbeing:	14 (31.1)		14 (37.8)	
Childcare,<br>approx. total personal cost (£), last 6 months		11.80 (63.60)<br>[0 to 400]		40.70 (122.50)<br>[0 to 600]
Domestic support,<br>approx. total personal cost (£), last 6 months		60.00 (307.00)<br>[0 to 1800]		18.50 (94.10)<br>[0 to 480]
Private physiotherapy.<br>approx. total personal cost (£), last 6 months		12.50 (55.30)<br>[0 to 300]		1.50 (7.70)<br>[0 to 40]
Private counselling,<br>approx. total personal cost (£), last 6 months		20.30 (88.80)<br>[0 to 500]		11.70 (47.90)<br>[0 to 240]
Massage,<br>approx. total personal cost (£), last 6 months		21.30 (51.70)<br>[0 to 210]		63.30 (179.00)<br>[0 to 720]
Fitness classes,<br>approx. total personal cost (£), last 6 months		25.10 (87.50)<br>[0 to 500]		21.80 (58.60)<br>[0 to 240]
Other:<br>Acupuncture<br>Osteopath<br>Chiropractor<br>Slimming World<br>Spa day<br>Gym/swim membership<br>Art classes	 0<br>0<br>1<br>1<br>1<br>1<br>1		 1<br>1<br>0<br>0<br>0<br>0<br>0	
Needed to stay off work, last 6 months	10 (23.8)		12 (34.3)	
Days off work, last 6 months		19.6 (52.7)<br>[0 to 182]		11.2 (32.0)<br>[0 to 130]

Child services/resources	**n (%)**	**Group mean (SD) [range]**	**n (%)**	**Group mean (SD) [range]**
Direct payments	16 (36.4)		11 (31.4)	
Use of direct payments:				
Enabler/Enabling service	7		6	
Overnight respite	3		1	
Behaviour support meetings	1		0	
In Touch Minehead learning group	0		1	
Jumpstart group	0		1	
Autism group	0		1	
Shared Lives	1		0	
Lifeworks activities	1		0	
Holiday club	2		0	
Horse therapy	0		1	
Autism assistance dog	0		1	
Hydrotherapy	0		1	
Massage	0		1	
Horse riding	1		0	
Swimming lessons	1		1	
Dance lessons	0		1	
Sensory music group	1		0	
Tutoring	0		1	
Cost of resources/services paid for with direct payments		852.10 (2561.00) [0 to 12000]		376.00 (880.20) [0 to 3000]
Child Social worker/Care manager appointments	7 (19.4)	0.9 (3.5) [0 to 20]	8 (22.9)	0.6 (2.1) [0 to 12]
Child support group sessions	6 (16.2)	1.6 (5.3) [0 to 30]	10 (28.6)	3.6 (12.5) [0 to 72]
Enabling service, hours in last 6 months	4 (12.1)	0.2 (0.6) [0 to 3]	7 (20.6)	6.3 (16.8) [0 to 72]
Short breaks, nights in last 6 months	7 (19.4)	2.1 (10.3) [0 to 60]	5 (14.3)	1.0 (3.2) [0 to 12]
Crisis respite care, nights in last 6 months	0		0	
Other child services/resources:				
CAMHS	2		0	
Mentor	1		0	
Lifeworks charity	1		0	
Safe Families Worker - crafts	0		1	
Phab Club	1		0	
Jam Buddies	1		0	
School Counsellor	1		0	
Other services/resources used or things done in last 6 months that improved health/wellbeing	**n (%)**		**n (%)**	

	7 (21.9)		7 (22.6)	
Gym classes	0		2	
Running	0		1	
Walking	0		1	
Weight loss coach	0		1	
Fibromyalgia support group	1		0	
IPSEA Advice line	0		1	
Spa break	1		1	
Weekend away with ASC partners and children met online	0		1	
CAMHS parents workshop	1		0	
Counselling for child	0		1	
Netball	1		0	
Karate	1		0	
Antidepressants	1		0	
Respite stay	1		0	
Online communities for hobbies	1		0	
Giving to others	0		1	
